# Endogenous luteinizing hormone concentration and IVF outcome during ovarian stimulation in fixed versus flexible GnRH antagonist protocols: An RCT

**Published:** 2018-03

**Authors:** Raffaella Depalo, Paolo Trerotoli, Annarosa Chincoli, Margherita Patrizia Vacca, Giuseppina Lamanna, Ettore Cicinelli

**Affiliations:** 1 *Department of Obstetrics-Gynecology-Neonatology and Anesthesiology, Unit of Medically Assisted Reproduction and Gametes Cryopreservation, University Hospital of Bari, Bari, Italy.*; 2 *Department of Biomedical and Human Oncological Science (DIMO), 2* ^*nd*^ * Unit of Obstetrics and Gynecology, University of Bari, Bari, Italy.*; 3 *Consultant Gynaecologist and IVF Specialist, London Women's Clinic, London, UK. *

**Keywords:** In vitro fertilization, Luteinizing hormone, Ovarian stimulation, Outcome

## Abstract

**Background:**

Luteinizing hormone (LH) is essential for normal follicular development and oocyte maturation. In particular, fluctuations of LH during the follicular phase have a significant impact on morphological and functional changes of the oocyte and determine its meiotic status and ability to be fertilized.

**Objective:**

This prospective randomized controlled trial examined effects of endogenous follicular phase LH levels on oocyte maturity and IVF outcomes in fixed vs. flexible in vitro fertilization.

**Materials and Methods:**

Normo-ovulatory women age <39 yr (n=213) were randomized to fixed or flexible gonadotrophin-releasing hormone (GnRH) antagonist protocols. Follicular phase LH, estradiol, and progesterone profiles were measured. Oocytes retrieved, implantation rate, and pregnancy rate were compared between the two groups.

**Results:**

LH profiles were similar in both protocols. A lower trend of LH values at the end of ovarian stimulation correlated significantly with a higher pregnancy rate, regardless of protocol (p=0.02). Estradiol levels were statistically different with respect to time points within treatment groups (p<0.0001), but not between groups (p=0.43), or pregnancy outcomes (p=0.2595). Progesterone profiles were similar between groups. No differences were found in retrieved oocytes numbers, fertilization rate or embryos obtained. Significantly, younger age and a higher number of antral follicles were correlated with positive results.

**Conclusion:**

Fixed and flexible GnRH antagonist protocols did not produce an oscillation of endogenous LH values correlated to the outcome of ovarian stimulation.

## Introduction

Luteinizing hormone (LH) is essential for normal follicular development and oocyte maturation. In particular, fluctuations of LH during the follicular phase have a significant impact on morphological and functional changes of the oocyte and determine its meiotic status and ability to be fertilized (1). The role of LH in controlled ovarian hyperstimulation (COH) and its relative importance during the follicular phase are still subject to extensive debate, and questions surrounding the optimal amount of LH in stimulation protocols are still controversial (2). 

Gonadotropin-releasing hormone (GnRH) antagonists have been used to inhibit a premature LH surge during ovarian stimulation for in vitro fertilization (IVF). GnRH antagonism is initiated following either a fixed scheme on day 6 of stimulation or a flexible scheme when ultrasound reveals follicles ≥14 mm in diameter (3-5). 

This provides a diverse hormonal milieu during early follicular recruitment, as there is no initial pituitary suppression. The optimal protocol for routine clinical use has not yet been identified. In fact, in a fixed regimen, a number of patients exhibit rapid and early follicular growth or elevated LH and estradiol (E2) levels prior to antagonist administration, due to positive feedback between LH and increasing levels of E2 during the early follicular phase. Thus, starting GnRH antagonist therapy in the middle of the follicular phase may be too late in some patients, because high LH levels during the follicular phase are associated with poor oocyte/embryo quality, with impaired endometrial receptivity and consequently, with a negative impact on IVF outcome (6, 7). 

On the other hand, some patients have elevated LH and low E2 levels with slow follicular growth prior to the administration of GnRH antagonist (8). In these women, early initiation of GnRH antagonist may reduce the ovarian response, leading to a suboptimal outcome of the IVF cycles. In both populations of patients, the endocrine environment of the early follicular phase in antagonist cycles is likely to influence IVF outcomes. 

The aim of the present study was to prospectively investigate how endogenous follicular phase LH levels in fixed vs. flexible GnRH antagonist protocols affect oocyte maturity and IVF outcomes.

## Materials and methods

In this randomized controlled trial, the random allocation was performed using computer-generated random numbers blocked into groups of 10 to reduce the chance that more patients in one group would be randomized in one time period during the study. A research nurse coordinated randomization and distribution of medication throughout the treatment cycles. 

**Participants**

Between May 2013 and May 2015, women undergoing ovarian stimulation and IVF with or without intracytoplasmic sperm injection were recruited at the Bari University Hospital Centre for Reproductive Medicine, and allocated to fixed or flexible GnRH antagonist protocols. Patients could been enrolled only once. Eligibility criteria were: 1) age <39 yr; 2) menstrual cycle of 26-32 days; 3) baseline follicle-stimulating hormone levels <12 IU/ml; 4) body mass index between 18-30 Kg/m^2^; 5) no polycystic ovaries; 6) no oral contraceptives in the last year, 7) normal partner spermogram according to 2010 World Health Organization (WHO) criteria.

**Treatments**

Recombinant follicle stimulating hormone (FSH; Gonal-f®, Merck Serono or Puregon®, MSD, Italy) and GnRH antagonist (Cetrotide®, Merck Serono or Orgalutran®, MSD, Italy) were used for COH with a starting recombinant FSH dose of 150 UI beginning on day 2-3 of the menstrual cycle and a daily dose of rFSH based on response. From day 6 of stimulation onward, GnRH antagonist was administered daily in the fixed protocol. In the flexible group 0.25 mg of GnRH antagonist was started when at least one of the following criteria was met: the presence of a follicle with mean diameter ≥14 mm, serum LH level ≥200 pg/mL per dominant follicle, LH ≥10 IU/L. 

GnRH antagonist was administered daily in both protocols until initiation of human chorionic gonadotrophin (hCG). Final oocyte maturation was triggered with 6500 IU of recombinant hCG (Ovitrelle®, Merck Serono, Geneva, Switzerland) when three or more follicles reached ≥18 mm in diameter. Oocytes were retrieved 36 hr later. The standard IVF procedures followed (5). One or two embryos were transferred under ultrasound guidance on day 2 or 3 of embryo culture. For luteal phase support, all patients received a daily dose of 400 mg of vaginal micronized progesterone (Progeffik®, Effik Italia). 

**Measurements**

Serum LH, progesterone (P) and estradiol (E2) levels were measured on day one of ovarian stimulation (D1), on the day after the administration of GnRH antagonist (D2) and on the day of hCG administration (D3). We evaluated LH, E2 and progesterone profiles by treatment group and pregnancy outcome. 

Samples were analyzed by a central laboratory at the University Hospital of Bari using ADVIA Centaur and ADVIA Centaur XP systems standardized against the second international WHO standard. The primary endpoint of this study was different in the levels of LH between groups, while the secondary endpoints were differences in the total number of retrieved oocytes and in the proportion of mature oocytes (MII) between groups, number of days and total dose of rFSH administered, and ongoing pregnancy rate defined as the presence of fetal heart activity at 12 wk on ultrasound scan after embryo transfer. Premature LH surge was defined as a serum LH level of ≥10 mIU/mL or an increase of more than 2.5 times the basal level (8). 

**Ethical consideration**

The study was approved by the Institutional Ethical Board of Bari University Hospital (St.2672, Ce.2346, De.6441) and written informed consent was obtained from each participants.

**Statistical analysis**

Continuous data were summarized as mean and standard deviation if the distribution was Gaussian, otherwise as a median and interquartile range. Simple comparisons between independent groups (such as treatment or pregnancy outcome) were performed with Student’s t or Wilcoxon tests as appropriate. Trends in estradiol, progesterone and LH levels were assessed using an ANOVA model for the repeated measure at baseline, day 6 (or oocyte diameter ≥14 mm), and hCG administration. In this analysis of variance for repeated measures, values were transformed to better approximate a Gaussian distribution. 

Counting variables (number of oocytes, number of mature oocytes, and number of inseminated and fertile oocytes) were also transformed, to analyze differences related to both treatment and pregnancy outcome. Results are presented as back-transformed values of means and 95% confidence limits. Percentage of transferred women and percentage of pregnant women are reported and compared for independent groups through chi-square test. All analyses were performed with 9.3 SAS®/IntNet software for personal computer and a value <0.05 was considered as statistically significant. 

Power and sample size calculations for comparison of LH profiles between groups, accounting for pregnancy outcome, were performed with G*Power software, considering 100 patients for each treatment group as maximum recruitment for the center and using an ANOVA model for the repeated measure and four groups (permutations of 2 protocols and 2 possible outcomes). We assumed that effect size could vary between 0.2 and 0.3. Thus a total sample size of 200 patients with the assumed effect size would yield a power between 0.8 and 0.9.

## Results

Of 213 women enrolled, 104 were allocated to the fixed group and 109 to the flexible group (mean age 35.0±3.7 vs. 35.4±3.2 yr). Two participants (one in each group) withdrew consent, 6 did not respond to gonadotropin stimulation and 3 were excluded because protocols were not followed correctly, leaving 101 women in each group (Figure I). Mean values for baseline characteristics in fixed vs. flexible protocol groups were similar (Table I).

There were statistically significant differences in LH levels at all time points (F=43.41, p<0.0001; Figure 2A). Regarding the E2 profile, the model suggests that E2 levels were statistically different for treatment group (F=413.06, p<0.0001), but not for pregnancy outcome (F=1.36, p=0.2595); neither treatment appears to be a significant factor (F=0.62, p=0.4314) (Figure 2B). Progesterone levels at each time point according to treatment protocol and pregnancy outcome are presented in figure 2C. 

Pregnancy IVF outcome was a statistically significant factor influencing LH profile (F=3.73, p=0.0261) (Figure 2A); whereas, protocol treatment group was marginally significant (F=4.23, p=0.0411). The ANOVA model suggests that the difference in estradiol levels was statistically different with respect to time point, but not to pregnancy outcome neither treatment appear to be a significant factor (Figure 2B). The progesterone profile was statistically different both for time point and pregnancy outcome; however, there was no difference between treatment groups (Figure 2C).

Outcome measures were the total number of oocytes, number of mature oocytes (meiosis II), fertilization rate, implantation rate and pregnancy rate. Results are reported in table III. The model for the analysis oocyte numbers was statistically significant, but only positive pregnancy outcome was a significant factor, while protocol group and the interaction between protocol group and pregnancy outcome was not significant. The mean total number of oocytes in women who became pregnancy was similar between fixed protocol group and flexible protocol group (Table III)

Mean duration of ovarian stimulation had been significantly longer in the fixed protocol group with respect to flexible one and in women who became pregnant. The total rFSH dose administered was significantly higher with the fixed protocol, but this was not significant with respect to outcome (Table II). A small but significant difference in the total dose of rFSH (IU) was administered in the flexible protocol (Table II). Premature luteinization was observed in six patients in the fixed protocol group (5.9%) and in nine patients in the flexible protocol group (8.9%). Successful transfers (n=184) were evenly distributed between groups (94% with the fixed protocol vs. 88.1% with the flexible protocol; (*x*^2^=2.2, p=0.1384). Pregnancy rates were similar between groups [35.8% (34/95) in the fixed group vs. 29.2% (26/89) in the flexible group; *x*^2^=0.9042, p=0.3416]. Stratification by pregnancy outcome revealed a significantly higher number of oocytes recruited, mature (metaphase II) oocytes, fertilization rate, cleavage rate and implantation rate in the pregnant group, regardless of protocol (Table III). In this study, there were no adverse effects in either group. 

**Table I:** Main baseline characteristic of participants respect to protocol

	**Fixed** **GnRH antagonist protocol**	**Flexible** **GnRH antagonist protocol**	**p-value**
Age (yr)*	35.35	35.44	0.8755
BMI (kg/m^2^)*	23.64	22.84	0.1559
AFC (number)*	11.51	11.47	0.955
FSH (pg/ml)*	7.132	8.0516	0.0616
P (pg/ml)*	0.72	0.59	0.3772
LH (pg/ml)*	4.31	4.31	0.3939
E2 (pg/ml)**	29.08	37.32	0.0292

BMI: Body mass index

AFC: Antral follicle count

FSH: Follicle stimulating hormone

P: Progesterone

LH: luteinizing hormone

E2: Estradiol

* Data presented as mean

** Data presented as medianp value from χ square test

**Table II:** Outcome of ovarian stimulation with fixed vs. flexible GnRH antagonist protocols

	**Fixed GnRH antagonist protocol**		**Flexible** **GnRH antagonist protocol**		**p-value**
Duration of stimulation (days)*	10.48	1.191	9.544	2.042	0.0009
Gonadotropins (UI)*	2475	1800-3000	2025	1575-3000	0.0398
GnRH antagonist (ampoules)*	4.55	1.72	3.75	1.15	<0.0001
Cases with LH >10 IU/mL (n/total)*	5.95		3/89		0.7217#
Follicles >14 mm at start GnRH antagonism (n)**	2	1-3	2	2-3	0.22
Follicle >18 mm at start hCG (n)**	2	1-3	2	1-3	0.72

p-value from multiple comparisons of ANOVA model.

# Fisher exact test

GnRH: Gonadotropin-releasing hormone

LH: Luteinizing hormone

hCG: Human chorionic gonadotropin

* Data presented as mean and SD.

** Data presented as median and interquartile.

**Table III:** Results of the main outcome measure regarding the total number of oocytes, mature oocytes, implanted oocytes, fertilization rate and implantation rate respect to fixed or flexible treatment protocol and pregnancy outcome

	**Treatment protocol**			**Pregnancy IVF outcome**		
	**Fixed** **GnRH antagonist**	**Flexible** **GnRH antagonist**	**p-value**	**Positive**	**Negative**	**p-value**
	**Mean (95% CI)**	**Mean (95% CI)**		**Mean (95% CI)**	**Mean (95% CI)**	
Total oocytes*	4.8 (4.1-5.7)	4.6 (3.9-5.4)	5.220	6.4 (5.6-7.3)	4.1 (3.6-4.7)	<0.0001
Mature oocytes (MII)*	3.85 (3.3-4.4)	3.85 (3.3-4.4)	5.190	5.4 (4.8-6.1)	3.2 (2.8-3.7)	<0.0001
Inseminated oocytes*	3.75 (3.20-4.37)	3.24 (2.83-3.69)	1.148	5.01 (4.35-5.75)	2.91 (2.55-3.30)	<0.0001
Fertilization rate**	0.7 (0.3-1)	0.8 (0.4-1)	0.580	1 (0.6-1)	0.7 (0.3-1)	0.0023
Implantation rate**	0 (0-0.3)	0 (0-0.25)	0.240	0.5 (0.3-0.5)	0	<0.0001

MII: Meiosis II.

* Data from Analysis of Variance

** Interquartile range in parentheses; data analyzed with the non-parametric test.

**Figure 1:**
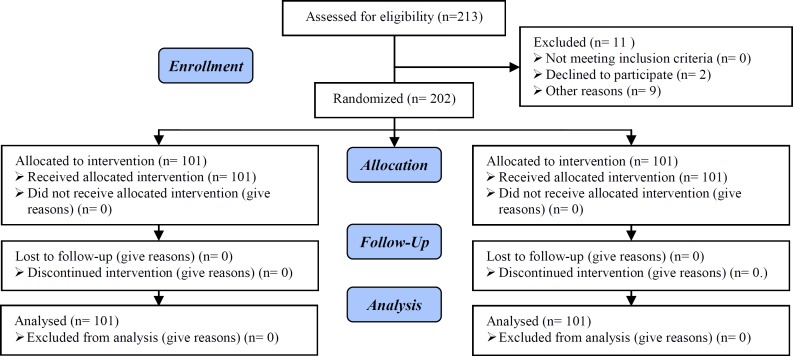
Flow chart about patients’ enrollment and data analisys

**Figure 2:**
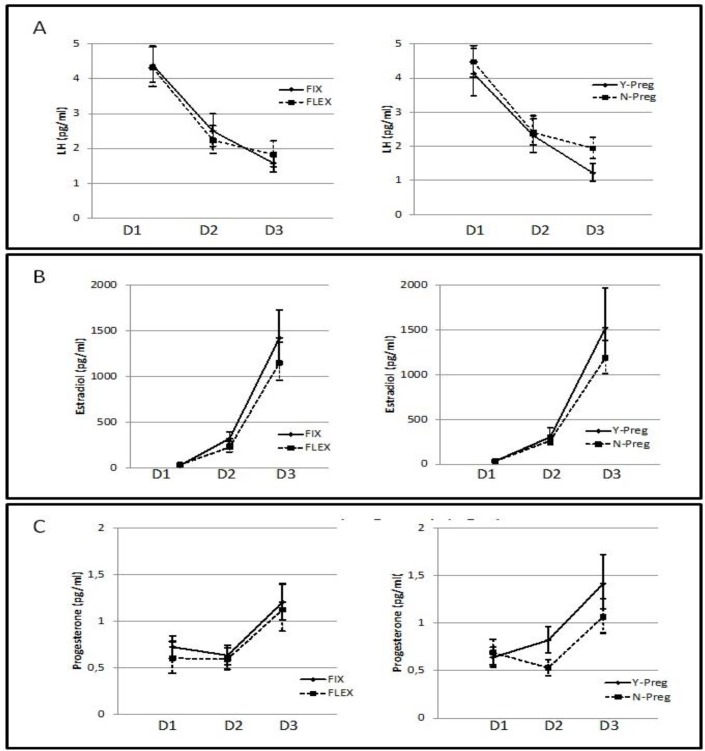
Hormone levels according to the treatment group and pregnancy status. (A) Luteinizing hormone profiles over the course of therapy in fixed vs. flexible protocols (right), and in pregnant vs. not pregnant women (left). In the fixed protocol group LH levels were 4.31 (95% CI 3.89-4.94) at baseline, 2.49 (95% CI 2.06-2.99) at day six and 1.58 (95% CI 1.32-1.86) at the end of stimulation; in flexible protocol the levels were 4.31 (95% CI 3.77-4.91) at baseline, 2.23 (95% CI 1.86-2.65) at day six and 1.82 (95% CI 1.47-2.22) at the end of stimulation. In women with positive pregnancy outcomes, the mean LH level was 4.12 (95% CI 3.48-4.86) at baseline, 2.31 (95% CI 1.82-2.89) at day six and 1.22 (95% CI 0.97-1.49) at the end of stimulation. (B) Estradiol profiles over the course of therapy in women following fixed protocol vs. flexible protocols (left), and in pregnant vs. not pregnant women (right). (C) Progesterone profile over the course of therapy in fixed vs. flexible protocols (left), and in pregnant vs. not pregnant women (right). D1, baseline; D2, day after initiating GnRH antagonism; D3, the day of hCG administration. In women with positive pregnancy outcomes, the value was 0.64 (95% CI 0.54-0.74) at baseline, 0.81 (95% CI 0.68-0.9) at day six, and 1.41 (95% CI 1.14-1.72) at the end of stimulation; in women without pregnancy, the progesterone level was 0.69 (95% CI 0.56-0.83) at baseline, 0.53 (95% CI 0.44-0.62) at day 6, and 1.07 (95% CI 0.89-1.25) at the end of stimulation. There was no difference between treatment groups (F=0.07, p=0.7877).

## Discussion

In fixed GnRH antagonist protocols, higher levels of LH and an earlier rise in E2 have been observed. However, some patients have an unexplained poor ovarian response with slow follicular growth and low serum E2 levels before GnRH antagonist administration. Thus, the endocrine environment may vary in the early follicular phase at the time of GnRH antagonist administration (4-13). 

Our findings confirm the importance of LH levels for COH outcomes. Pregnancy outcome was significantly influenced by the LH profile, with women who became pregnant having significantly lower LH levels at baseline and at the end of stimulation (F=3.73, p=0.0261) (Figure 1A). Previous research has indicated that fluctuations in LH levels during the follicular phase may be detrimental to endometrial receptivity and hence to pregnancy rates (2, 6). Moreover, when we stratified the population into pregnant and non-pregnant patients, we observe that lower LH levels at baseline and at the initiation of hCG are associated with positive outcomes. Conversely, the GnRH antagonist protocol did not appear to play a major role. In agreement with previous studies, (14-17) our data demonstrate that the timing of GnRH antagonism does not affect the result of the cycle. 

We found that the total number of oocytes, number of mature oocytes, fertilization and implantation rates were higher in women who became pregnant. In agreement with previous studies, we found that the ovarian response to COH and the IVF outcome was dependent on patient age and the pool of recruitable follicles, as well as on follicular sensitivity to FSH (18, 19). Indeed, considering the baseline characteristics in pregnant and non-pregnant women, we observe that the outcome depends on patient age and antral follicle count. 

We evaluated also the adverse effects of premature LH surge during the follicular phase. In our study, the incidence of premature LH rise was similar between the two protocols, which is in agreement with a previous report (15). There were no significant differences in stimulation characteristics between the two study groups. A small but significantly longer mean length of ovarian stimulation was observed in women who became pregnant, whereas there were no differences in gonadotropin consumption.

Concerning the E2 profile, our data did not reveal a difference between the two treatment protocols. E2 concentrations were higher at each time point in women with positive outcomes, but the difference was not statistically significant. Both protocols are able to maintain low concentrations of progesterone for the duration of the stimulation and this is important to avoid premature luteinization, which could reduce the pregnancy rate by prematurely closing the window for implantation. Moreover, we observed a significant difference between pregnant and non-pregnant women at each time point, with a progressive linear increase in progesterone levels during ovarian stimulation in women with positive pregnancy outcomes. There is a growing consensus that high P levels at the end of the follicular phase are detrimental to clinical outcome (14, 20-22). Our findings, in agreement with those of Griesinger *et al* allow us to hypothesize that P levels above a threshold of 1.5 ng/mL are independently associated with a decreased probability of pregnancy in normal responders (17, 20).

## Conclusion

In conclusion, this study has demonstrated that a lower trend for LH concentrations from baseline to the end of the ovarian stimulation is related to a higher pregnancy rate, regardless of the type of GnRH antagonist protocol used. Oocyte number and maturity are not influenced by GnRH antagonist protocols, but lower age and a larger pool of recruitable follicles predict successful impregnation.

## Note

***Registration ID in Trial Registration: NCT02328924***

## References

[1] Raju GA, Chavan R, Deenadayal M, Gunasheela D, Gutgutia R, Haripriya G (2013). Luteinizing hormone and follicle stimulating hormone synergy: A review of role in controlled ovarian hyper-stimulation. J Hum Reprod Sci.

[2] Huirne JA, van Loenen AC, Schats R, McDonnell J, Hompes PG, Schoemaker J (2005). Dose-finding study of daily GnRH antagonist for the prevention of premature LH surges in IVF/ICSI patients: optimal changes in LH and progesterone for clinical pregnancy. Hum Reprod.

[3] Ganirelix Dose-finding Study Group (1998). A double-blind, randomized, dose finding study to assess the efficacy of the gonadotrophin-releasing hormone antagonist ganirelix (Org 37462) to prevent premature luteinizing hormone surges in women undergoing ovarian stimulation with recombinant follicle stimulating hormone (Puregon) The ganirelix dose-finding study group. Hum Reprod.

[4] Copperman AB, Benadiva C (2013). Optimal usage of the GnRH antagonists: a review of the literature. Reprod Bio Endocrinol.

[5] Depalo R, Jayakrishan K, Garruti G, Totaro I, Panzarino M, Giorgino F (2012). GnRH agonist versus GnRH antagonist in in vitro fertilization and embryo transfer (IVF/ET). ReprodBiolEndocrinol.

[6] Kolibianakis EM, Albano C, Kahn J, Camus M, Tournaye H, Van Steirteghem AC (2003). Exposure to high levels of luteinizing hormone and estradiol in the early follicular phase of gonadotropin-releasing hormone antagonist cycles is associated with a reduced chance of pregnancy. Fertil Steril.

[7] Bosch E, Escudero E, Crespo J, Simón C, Remohí J, Pellicer A (2005). Serum luteinizing hormone in patients undergoing ovarian stimulation with gonadotropin-releasing hormone antagonists and recombinant follicle-stimulating hormone and its relationship with cycle outcome. Fertil Steril.

[8] Sönmezer M, Pelin Cil A, Atabekoğlu C, Ozkavukçu S, Ozmen B (2009). Does premature luteinization or early surge of LH impair cycle outcome? Report of two successful outcomes. J Assist Reprod Genet.

[9] Depalo R, Garruti G, Totaro I, Panzarino M, Vacca MP, Giorgino F (2011). Oocyte morphological abnormalities in overweight women undergoing in vitro fertilization cycles. Gynecol Endocrinol.

[10] Kolibianakis EM, Zikopoulos K, Schiettecatte J, Smitz J, Tournaye H, Camus M (2004). Profound LH suppression after GnRH antagonist administration is associated with a significantly higher ongoing pregnancy rate in IVF. Hum Reprod.

[11] Lainas T, Zorzovilis J, Petsas G, Stavropoulou G, Cazlaris H, Daskalaki V (2005). In a flexible antagonist protocol, earlier, criteria-based initiation of GnRH antagonist is associated with increased pregnancy rates in IVF. Hum Reprod.

[12] Kolibianakis EM, Albano C, Camus M, Tournaye H, Van Steirteghem AC, Devroey P (2003). Initiation of gonadotropin-releasing hormone antagonist on day 1 as compared to day 6 of stimulation: effect on hormonal levels and follicular development in in vitro fertilization cycles. J Clin Endocrinol Metab.

[13] Merviel P, Antoine JM, Mathieu E, Millot F, Mandelbaum J, Uzan S (2004). Luteinizing hormone concentrations after gonadotropin-releasing hormone antagonist administration do not influence pregnancy rates in in vitro fertilization-embryo transfer. Fertil Steril.

[14] Griesinger G, Shapiro DB, Kolibianakis EM, Witjes H, Mannaerts BM (2011). No association between endogenous LH and pregnancy in a GnRH antagonist protocol: part II, recombinant FSH. Reprod Biomed Online.

[15] Al-Inany H, Aboulghar MA, Mansour RT, Serour GI (2005). Optimizing GnRH antagonist administration: meta-analysis of fixed versus flexible protocol. Reprod Biomed Online.

[16] Hamdine O, Broekmans FJ, Eijkemans MJ, Lambalk CB, Fauser BC, Laven JS (2013). Early initiation of gonadotropin-releasing hormone antagonist treatment results in a more stable endocrine milieu during the mid- and late-follicular phases: a randomized controlled trial comparing gonadotropin-releasing hormone antagonist initiation on cycle day 2 or 6. Fertil Steril.

[17] La Marca A, Grisendi V, Giulini S, Argento C, Tirelli A, Dondi G (2013). Individualization of the FSH starting dose in IVF/ICSI cycles using the antral follicle count. J Ovarian Res.

[18] Papanikolaou EG, Pados G, Grimbizis G, Bili E, Kyriazi L, Polyzos NP (2012). GnRH-agonist versus GnRH-antagonist IVF cycles: is the reproductive outcome affected by the incidence of progesterone elevation on the day of HCG triggering? A randomized prospective study. Hum Reprod.

[19] Griesinger G, Mannaerts B, Andersen CY, Witjes H, Kolibianakis EM, Gordon K (2013). Progesterone elevation does not compromise pregnancy rates in high responders a pooled analysis of in vitro fertilization patients treated with recombinant follicle-stimulating hormone/gonadotropin-releasing hormone antagonist in six trials. Fertil Steril.

[20] Detti L, Yelian FD, Kruger ML, Diamond MP, Rode A, Mitwally MF (2008). Endometrial thickness is related to miscarriage rate, but not to the estradiol concentration, in cycles down-regulated with gonadotropin-releasing hormone antagonist. Fertil Steril.

[21] Huirne JA, Homburg R, Lambalk CB (2007). Are GnRH antagonists comparable to agonists for use in IVF?. Hum Reprod.

